# Exploring Thermal Sensitivities and Adaptations of Oxidative Phosphorylation Pathways

**DOI:** 10.3390/metabo12040360

**Published:** 2022-04-17

**Authors:** Hélène Lemieux, Pierre U. Blier

**Affiliations:** 1 Faculty Saint-Jean, Department of Medicine, Faculty of Medicine and Dentistry, University of Alberta, Edmonton, AB T6C 4G9, Canada; 2 Department Biologie, Université du Québec à Rimouski, Rimouski, QC G5L 3A1, Canada

**Keywords:** mitochondrial function, thermal sensitivity, NADH pathway, succinate pathway, electron-transferring flavoprotein, glycerophosphate dehydrogenase, dihydroorotate dehydrogenase, choline dehydrogenase, proline dehydrogenase, sulfide:quinone oxidoreductase

## Abstract

Temperature shifts are a major challenge to animals; they drive adaptations in organisms and species, and affect all physiological functions in ectothermic organisms. Understanding the origin and mechanisms of these adaptations is critical for determining whether ectothermic organisms will be able to survive when faced with global climate change. Mitochondrial oxidative phosphorylation is thought to be an important metabolic player in this regard, since the capacity of the mitochondria to produce energy greatly varies according to temperature. However, organism survival and fitness depend not only on how much energy is produced, but, more precisely, on how oxidative phosphorylation is affected and which step of the process dictates thermal sensitivity. These questions need to be addressed from a new perspective involving a complex view of mitochondrial oxidative phosphorylation and its related pathways. In this review, we examine the effect of temperature on the commonly measured pathways, but mainly focus on the potential impact of lesser-studied pathways and related steps, including the electron-transferring flavoprotein pathway, glycerophosphate dehydrogenase, dihydroorotate dehydrogenase, choline dehydrogenase, proline dehydrogenase, and sulfide:quinone oxidoreductase. Our objective is to reveal new avenues of research that can address the impact of temperature on oxidative phosphorylation in all its complexity to better portray the limitations and the potential adaptations of aerobic metabolism.

## 1. Introduction

Anticipated global climate changes will be a major threat to ectothermic organisms and the overall biodiversity of the planet [1]. For different species, the effects of these environmental alterations will depend on the range of temperatures they can tolerate (thermal tolerance), the impact of the temperature changes on their physiological performance and life history traits, and their ability to adjust to new environmental conditions. Therefore, the thermal sensitivity of different physiological functions, and their capacity to compensate for increased temperatures, might dictate the long-term consequences of global climate changes on their survival.

The bioenergetics of organisms—particularly cellular bioenergetics upstream of a complex physiological function—is affected by temperature [2]. Changes in temperature, therefore, necessarily impact the whole organism through the thermal sensitivity of cellular bioenergetics machinery, particularly the mitochondria [3]. There is also some evidence that impairment of the mitochondrial structure or function might set the upper thermal limits of ectotherms [4,5]. Furthermore, temperature acclimation is associated with changes in mitochondrial membrane composition that could lead to changes in enzyme activities [2].

Documenting to what extent OXPHOS is affected by temperature in different species from different thermal habitats could, therefore, be a simple way to assess mitochondrial adaptability. Another approach would be to identify the steps and pathways that are more affected by temperature and could impede ATP production or ROS management. The identification of these steps, combined with a delineation of divergences in mitochondrial architecture and organization in different species adapted to different thermal niches, could identify potential selection sites. Further studies on the genetic and phenotypic variability of these traits, as well as their heritability, are a prerequisite for estimating their adaptability and, therefore, the possibilities and constraints for the evolution of cellular bioenergetics. For example, studies on Drosophila have demonstrated that different mitochondrial haplotypes are associated with different thermal sensitivities [6,7].

Mitochondrial DNA encodes for important peptides of the ETS complexes and ATP synthase; these peptides are involved in the metabolic adaptations of organisms [8]. In combination with these mtDNA-encoded peptides, mitochondria also require hundreds of nuclear genes (proteins) to orchestrate the assembly of fully functional mitochondria. The identification of all genes and proteins involved in the potential adaptations of new phenotypes through genomic and proteomic approaches will be more complex than just comparing metabolic phenotypes associated with potential adaptation and mtDNA (see [9]). Accordingly, the identification of sensitive steps through extensive characterization of OXPHOS capacity when mitochondria are presented with different metabolites, inhibitors, and uncouplers at different temperatures [10], the measurement of changes in various species following thermal acclimation, and the comparison of species from different thermal habitats, are powerful and simpler approaches for identifying sensitive or plastic traits and loci that are likely under selective pressure.

**Figure 1:**
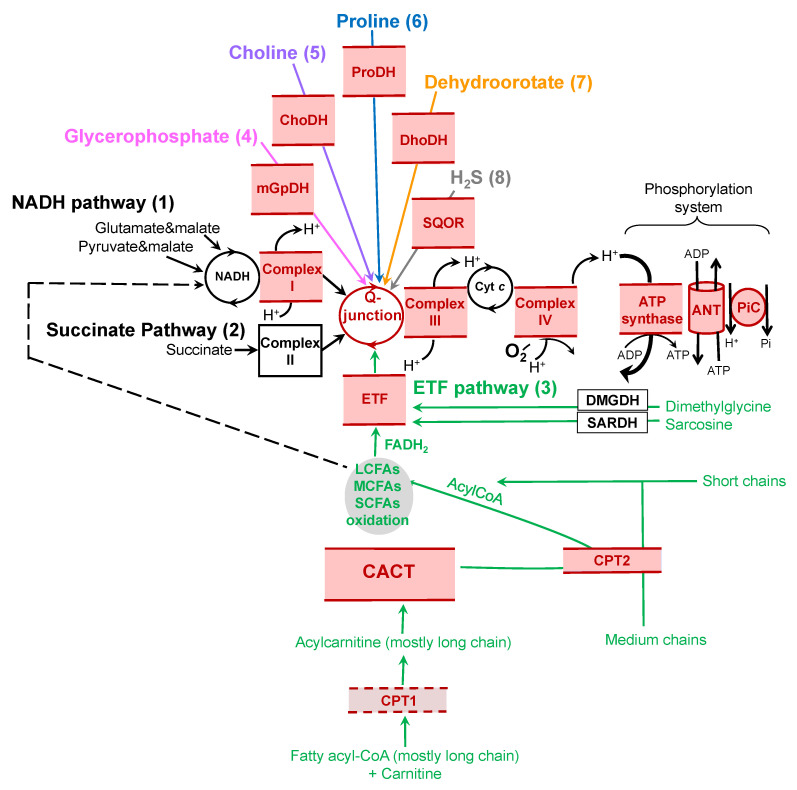
Electron entrance in oxidative phosphorylation (OXPHOS). The main entry points converging at the Q-junction (ubiquinol/ubiquinone) of the OXPHOS system are illustrated here and described as numbers 1 to 8. (1) The NADH pathway provides electrons to complex I of the electron transport system. These electrons can be provided—among others—by pyruvate (through pyruvate dehydrogenase), and glutamate (through glutamate dehydrogenase). (2) The succinate pathway uses succinate dehydrogenase to reduce FAD and feed complex II. (3) The electron-transferring flavoprotein (ETF) pathway (in light green) receives electrons from fatty acyl-CoA of various chain lengths. The carnitine shuttle system is composed of carnitine palmitoyltransferase 1 (CPT1), carnitine–acylcarnitine translocase (CACT), and carnitine palmitoyltransferase 2 (CPT2). Oxidation of the long-chain fatty acids (LCFAs) depends on the carnitine shuttle system, whereas the oxidation of short- (SCFAs) and medium- (MCFAs) chain fatty acids are largely independent of that system (reviewed by [12]). Once in the mitochondrial matrix, the fatty acids enter the process of long-chain, medium-chain, or short-chain fatty acid oxidation. In this process, NAD and FAD are reduced to NADH and FADH_2_; FADH_2_ is used as a substrate for electron-transferring flavoprotein (ETF), while NADH feeds complex I. Both NADH and FADH_2_ provide electrons into the Q-junction. The dashed line links LCFA, MCFA, and SCFA oxidation to the NADH pathway, which is not rate-limiting for the ETF pathway. Malate is also provided, with all fatty acid substrates, as a metabolite in the citric acid cycle to prevent the accumulation of acetyl-CoA and the concurrent inhibition of fatty acid oxidation. Dimethylglycine dehydrogenase (DMGDH) and sarcosine dehydrogenase (SARDH) can also feed electrons through the ETF pathway. (4) Glycerophosphate oxidation (in pink) occurs via mitochondrial glycerophosphate dehydrogenase (mGpDH) and shuttles electrons through FADH_2_ directly to the Q-junction. (5) Choline oxidation (in purple) occurs through choline dehydrogenase (ChoDH), which reduces FAD. (6) Proline dehydrogenase (ProDH, in blue) catalyzes proline oxidation and FAD reduction. (7) The dihydroorotate (Dho, in orange) is transformed into orotate by dihydroorotate dehydrogenase (DhoDH), releasing an electron from flavin mononucleotide directly into the Q-junction. (8) sulfide:quinone oxidoreductase (SQOR, in grey) oxidizes H_2_S and transfers an electron into the Q-junction. After convergence of electrons from diverse pathways at the Q-junction, they follow a linear segment through complexes III and IV before reducing molecular oxygen. Electron transfer is coupled with proton pumping into intermembrane space by complexes I, III, and IV, generating a proton motive force, and providing the energy required to phosphorylate ADP into ATP via ATP synthase (part of the phosphorylation system, also supported by the adenine nucleotide translocase, ANT, and the phosphate carrier, PiC). All components located in, or peripherally associated with, the mitochondrial membranes are in red; solid lines represent those associated with the inner membrane and the dashed line represents the one component associated with the outer mitochondrial membrane (i.e., CPT1).

We first need, however, to identify the relevant pathways used by different species or tissues and determine how these pathways react to short- (acute effects) and long- (adaptation) term variations in temperature. Traditionally, OXPHOS measurement was limited to the quantification of mitochondrial oxygen consumption after providing substrates for the reduction of complex I (NADH dehydrogenase; NADH pathway) or complex II (succinate dehydrogenase; succinate pathway), separately at first, and more recently in combination. These two pathways deliver electrons to the ubiquinone pool (Q-junction) that sequentially supplies complex III, cytochrome *c*, complex IV, and molecular oxygen. While studies on the thermal sensitivity of mitochondria focused on the NADH and succinate pathways in the last few decades, recent studies have concentrated on the combination of these two pathways, or have included other pathways, such as electron-transferring flavoprotein (ETF), mitochondrial glycerol-3-phosphate dehydrogenase (mGpDH), proline dehydrogenase (ProDH), choline dehydrogenase (ChoDH), dihydroorotate dehydrogenase (DhoDH), and sulfide:quinone oxidoreductase (SQOR) pathways (Figure 1). Recent progress has shown that these alternate pathways, in combination with canonic pathways, can result in significantly higher capacities to reduce the Q-junction (reviewed by [11]). It is likely that the evolution of these pathways, as well as their orchestration and combination, could lead to new metabolic adaptations. The aim of our paper is to review these understudied pathways and their thermal sensitivities, and to suggest rigorous approaches to include them when exploring potential adaptations of OXPHOS to environmental change.

## 2. NADH and Succinate Pathways

The most extensive literature available concerning the effect of temperature on mitochondrial function focuses on the NADH and succinate pathways, along with their closely associated steps. Some reviews are already available on these topics [2,3], and we will not add further details here. Briefly, the pyruvate dehydrogenase complex, a key step in pyruvate oxidation that feeds the NADH pathway, has been found to be largely responsible for respiratory limitation at low temperatures in warm-adapted species, such as rats [10]. Low-temperature respiratory limitation by pyruvate dehydrogenase has been overcome in cold-adapted species, such as the Atlantic wolffish (*Anarhichas lupus*) [13]. The importance of pyruvate dehydrogenase in regulating temperature preference is also supported by a study on a cold-seeking mutant of *Drosophila melanogaster*, which showed that the mutant with a preference for a cold environment had higher activity in the pyruvate dehydrogenase complex (PDC) [14]. Once the ectotherm species have overcome the limitation by PDC in the cold environment, complex IV thermal sensitivity becomes highly correlated with the thermal sensitivity of mitochondrial respiration; this relationship is not observed in endotherm species [2]. Furthermore, results from titrations of complex IV inhibitors at various temperatures in the red muscle of brook trout (*Salvelinus fontinalis*) suggest that complex IV exerts a high control coefficient on the ETS in the normal temperature range [15], but that the control of mitochondrial catalytic capacities might switch at lower temperatures to processes upstream of the ETS, such as the PDC [2].

Succinate and succinate dehydrogenase also contribute to electron transfer through FAD at the Q-junction, leading to an increased respiration rate. This additive respiration varies depending on species, strains, organs, and experimental conditions. In endotherms, the respiration rate following succinate addition to the mitochondria already fed at the complex I level is higher by 1.6- to 2.0-fold in rat heart [16,17], 1.2- to 1.8-fold in rat skeletal muscle [18,19,20], 1.4-fold in mouse skeletal muscle [21], and 1.3- to 2.1-fold in human skeletal muscle (reviewed by [22]). In fish heart mitochondria, the increase in OXPHOS resulting from the addition of succinate is rather variable, depending on species, time, and temperature. In salmon (*Salmo salar*) acclimated to 12 °C, the increase goes from 1.5-fold when measured at 20 °C, to approximately 2-fold when measured between 24 °C and 28 °C. When acclimated to 20 °C, the increase is lower, from 1.12-fold when measured at low temperatures, to 1.4-fold when measured at higher temperatures [23]. In European perch (*Perca fluviatilis*), the increment is lower compared to salmon and mammal tissues, and it is similar at two temperatures of measurement (i.e., from 1.10- to 1.12-fold at 16 °C and from 1.06- to 1.11-fold at 23 °C) [24]. In rainbow trout (*Oncorhynchus mykiss*) heart mitochondria, the increment was close to 1.2-fold between 5 °C and 30 °C [25] and between 1.1- and 1.2-fold in the wrasse (*Notolabrus celidotus*) when measured between 15 °C and 32.5 °C [26]. The magnitude of increment in respiratory capacity ensured by SDH activity is, therefore, species- and temperature-dependent, and is also modulated by acclimation temperature, at least in Atlantic salmon (*Salmo salar*). It has been reported that this reserve respiratory capacity, might be vital for cardiac myocytes [27], particularly for their ability to survive hypoxia [28], and potentially for their ability to survive temperature variation.

In the ETS, complexes I and III are known as the primary sites of ROS generation, with the suspected participation of SDH through the reverse flow of electrons to complex I generation sites [29,30,31,32]. It was later observed that SDH generates ROS through its FAD site [33,34,35]. Mitochondrial ROS management is of crucial importance for the governance of different pathways, not only in the aging process [36], but also for the tolerance of ectotherms to increased temperatures [4]. We can therefore speculate that the metabolic requirements of ROS could partly dictate ETS architecture, and particularly the relative quantity or activity of the different complexes [37]. In a comparative analysis of two species of bivalve (*Arctica islandica* and *Mya arenaria*), Munro et al. [38] observed a much lower rate of H_2_O_2_ efflux in the mitochondria of the long-lived species (*A. islandica*; maximum lifespan 507 years) compared to the short-lived species, which was also associated with considerably lower SDH activity relative to cytochrome *c* oxidase or citrate synthase. ROS management has been extensively associated with organism lifespans, and the activities of the major mitochondrial ROS producers (CI and CIII) have been associated with lifespan modulation (see [36,39] for reviews). These results, and the involvement of SDH in ROS regulation, call for a better portrayal of the relative contribution of these ETS complexes, including SDH, in future studies seeking to determine how mitochondria are precisely tailored and adapted to different environmental conditions.

## 3. Electron-Transferring Flavoprotein Pathway

The electron-transferring flavoprotein pathway (see details in Figure 1) is of major importance in many tissues and species. A few preliminary observations support the importance of fatty acid catabolism in adjustments of mitochondrial functions that occur during temperature variations. A recent study on zebrafish (*Danio rerio*) showed that (1) fasting enhances acute cold resistance, and (2) stimulation of lipid catabolism is associated with the improvement of cold resistance [40]. Interestingly, several observations in zebrafish have linked cold resistance to lipid metabolism, including (1) the inhibition of fatty acid ß-oxidation by the suppression of the mTOR pathway weakened fasting-induced cold resistance, (2) the stimulation of fatty acid ß-oxidation using fenofibrate increased survival rate following cold stress, and (3) the suppression of fatty acid ß-oxidation using mildronate caused a decrease in survival following cold stress [40]. These results clearly point out the importance of modulating fatty acid ß-oxidation for animal resistance to cold temperatures.

Exposure to cold temperatures has also been associated with changes in the activities of various enzymes involved in lipid metabolism in animals, especially carnitine palmitoyl transferase (CPT) and ß-hydroxyacyl-CoA dehydrogenase (HOAD), a mitochondrial matrix enzyme involved in ß-oxidation of fatty acids. For example, there is an increase in red muscle CPT1 in striped bass (*Morone saxatilis*) [41], and muscle HOAD in rainbow trout (*Oncorhynchus mykiss*) [42] and sea bream (*Sparus aurata*) [43], following cold acclimation. Similarly, the activities of muscle CPT1 and ß-hydroxyacyl-CoA dehydrogenase are increased in fish species from Antarctic regions compared to species from temperature zones [44], and in horse mussels (*Modiolus modiolus*) collected in winter compared to those collected in summer [45]. Higher muscle HOAD activity was also observed in cold-acclimated striped marsh frogs (*Limnodynastes peronii*) compared to warm-acclimated frogs [46]. Furthermore, another study revealed higher expression of genes linked to fatty acid metabolism, such as fatty acid binding protein 1 (FABP1) and/or acyl-coA binding protein (ACBP), in the liver and brain of carp (*Cyprinus carpio* L.) following cold adaptation [47]. These results are in line with a recent comparative study on the hearts of eight fish species collected over a wide environmental temperature gradient (optimal temperatures ranging from −1 °C to above 30 °C) that revealed much higher relative activities of CPT1 and HOAD (when normalized to citrate synthase activity) in cold-adapted fishes compared to warm-adapted ones [37]. Overall, these results suggest an increase in fatty acid oxidation when animals are exposed to cold temperatures.

Very few comparative studies on the impact of acute change of temperature on the metabolism of intact mitochondria have focused on fatty acid catabolism. Rare examples include a study on red muscle mitochondria from shorthorn sculpin (*Myoxocephalus scorpius* (Linnaeus, 1758)) [48]. This study demonstrated that the thermal sensitivity (expressed by the Q_10_) of palmitoylcarnitine oxidation, a long-chain fatty acid, is reduced by half after cold acclimation. However, mitochondrial respiration measurement with palmitoylcarnitine + malate as the only substrates cannot reveal at which step the modification occurs, i.e., adjustments in CACT, CPT2, long-chain fatty acid oxidation, or ETF can cause the observed compensation (Figure 1). To address this shortcoming, a recent study on the planarian *Dugesia tigrina* evaluated the impact of thermal acclimation on mitochondrial respiration using three sets of fatty acid ß-oxidation substrates: palmitoylcarnitine + malate, octanoylcarnitine + malate, and acetylcarnitine + malate (Mast and Lemieux, resubmitted). This allowed the assessment of the temperature effect on ß-oxidation for specific chain lengths (long- or medium-chain fatty acids, with palmitoylcarnitine or octanoylcarnitine, respectively) and on specific enzymes involved in the pathway, such as carnitine palmitoyltransferase 2 (CPT2; only required for palmitoylcarnitine and octanoylcarnitine oxidation), carnitine–acylcarnitine translocase (CACT; required for combinations of the three substrates), or carnitine acyltransferase (CAT; only required for acetylcarnitine oxidation). This study identified the medium-chain fatty acid oxidation capacity—but not the long-chain fatty acid oxidation capacity—as being adjusted following cold-acclimation in *D. tigrina*. To our knowledge, this is the only study that examines thermal sensitivity and the impact of thermal acclimation on multiple steps of fatty acid ß-oxidation in intact, functional mitochondria. More studies on substrate combinations in multiple tissues and different species are required to document the thermal sensitivity and plasticity of fatty acid oxidation and, therefore, the adaptability of the process.

Other mitochondrial enzymes that can sustain electron transport via ETF are enzymes involved in one-carbon metabolism, i.e., dimethylglycine dehydrogenase (DMGDH) and sarcosine dehydrogenase (SARDH) [49,50]. One-carbon metabolism includes the methionine and folate cycles, which provide one carbon unit (methyl group) during the synthesis of DNA, polyamines, amino acids, creatine, and phospholipids (for a review, see [51]). Both enzymes are also involved in choline metabolism. In mammalian liver cells, dimethylglycine and sarcosine are produced by choline oxidation [52,53,54]. Choline is involved in different physiological processes, including the transport of lipids, methylation reaction, neurotransmitter synthesis, and membrane phospholipid synthesis. Since choline is a micronutrient in different animal species, the function of DMGDH and SARDH in choline metabolism is oriented either to choline concentration adjustment, or to the production of sarcosine or glycine. Modulation of these enzymes is likely controlled by the requirements of anabolic or regulation pathways (regulation of choline, dimethylglycine, and sarcosine concentration and synthesis) rather than bioenergetic demands. Considering the role of choline in phospholipid synthesis, the modulation of the choline concentration by the regulation of DMGDH and SARDH activities could partly be involved in forming efficient mitochondrial membranes in ectotherms to face different thermal regimes. For example, mitochondrial respiratory capacity in the eurythermal teleost *Fundulus heteroclitus* is linked to the restructuring of membrane lipid composition via local adaptation as well as thermal acclimation [55].

## 4. Glycerophosphate Dehydrogenase

Mitochondrial glycerophosphate dehydrogenase (mGpDH, E.C. 1.1.99.5), found at the outer portion of the inner mitochondrial membrane [56], irreversibly oxidizes glycerol-3-phosphate to dihydroxyacetone phosphate (DHAP), feeding electrons from FADH_2_ into the Q-junction. The mGpDH functions together with the cytoplasmic form of the same enzyme (cGpDH, E.C. 1.1.1.8), which catalyzes the reverse conversion of DHAP into glycerol-3-phosphate (G3P), using NADH as the electron donor. In addition, mGpDH is also known as the rate-limiting enzyme for the glycerophosphate shuttle [57]. This enzyme shuttle, composed of mGpDH and cGpDH, is present in cells from a diverse range of animals, including insects [58], fish [59,60], yeast (see a review by [61]), and mammals [62,63,64,65,66].

A major function of mGpDH is likely the reoxidation of cytosolic NADH produced by glycolysis, and the transfer of reducing equivalents from the cytosol to the mitochondrial electron transport system [67]. However, its wide distribution in organisms, and its large variation at the tissue level, implies other functions [68]. The substrate for mGpDH—glycerol-3-phosphate—is, in fact, a pivotal metabolite that links carbohydrate to lipid metabolism. In addition to its link with OXPHOS, it can supply the carbon for gluconeogenesis and/or act as a backbone in triacylglycerol synthesis. It may also be provided as a substrate following the mobilization and lipolysis of acylglycerols, leading to free fatty acids and glycerol. This makes mGpDH a critical component in metabolism, being at the central junction between OXPHOS, glycolysis, and fatty acid metabolism [57,69,70,71,72]. Thus, it is not surprising that, changes in the expression of mGpDH have also been observed in several pathologies, such as cancer and metabolic diseases (reviewed by [68]). In recent studies, G3P has been proposed as a major substrate for mitochondrial respiration in some insects [73] and bivalves [74,75].

Very little work has been conducted on the impact of temperature on the GpDH shuttle; however, there is some indication that this enzyme system could be involved in the response to temperature changes. In a recent study on the yeast *Saccharomyces*, mGpDH activity was listed as one of 46 reactions important for growth at low temperatures [76]. A recent study showed that when various *Drosophila* species are held above their normal temperature range, NADH pathway (dependent on complex I) capacity is sharply reduced, but compensated for by a higher reliance on alternative pathways, such as mGpDH and ProDH, that act to maintain maximal OXPHOS capacity [77]. An increase in the expression of both cGpDH and mGpDH has been observed with increasing latitude (associated with decreasing temperatures) in Drosophila populations from southern Florida to Ontario [78]. This increase has been associated with the availability of nutrients along the latitudinal tropical–temperate gradient [78]. Thus, it seems that temperature along these gradients could lead to mitochondrial adjustments in pathway organization in ectotherms. Furthermore, mGpDH has been shown to mediate thermogenesis through modulation of activity in mouse brown adipose tissue following exposure to cold [79]. In these conditions, brown adipose tissue releases almost all the energy transduced by OXPHOS as heat via the uncoupling protein 1. The glycerophosphate shuttle supports brown adipose tissue thermogenesis by feeding additional reducing equivalents to the mitochondria [11]. In addition, knockout of mouse mGpDH leads to reduced energy turnover (food intake and oxygen consumption), increased thyroidal secretion, brown adipose tissue atrophy, and increased of uncoupling protein 3 when exposed to 32 °C, suggesting an attempt to compensate for a thermogenic deficiency [80].

Due to the fact that mGpDH function is affiliated with the cytosolic form cGpDH, we can expect that if one isozyme is affected by temperature change, the other would also be affected. There is some evidence that the cytosolic form of this enzyme is involved in the temperature acclimation of animal metabolism. First, the enzyme is activated following cold acclimation in endotherms. cGpDH in mice has been shown to greatly increase in the brown adipose tissue after exposure to cold conditions [81,82]. cGpDH gene expression also increases in the brain and liver of carp (*Cyprinus carpio* L.) following cold acclimation [47]. Second, cGpDH has been linked to resistance to cold damage. The loss of cGpDH decreases tolerance to freezing in wildtype *Saccharomyces cerevisiae* cells preincubated at low temperatures, but not in cells directly transferred from 30 °C to −20 °C [83]. This indicates that the mechanism behind the loss of tolerance requires time, and might be explained by processes dependent on cGpDH, but not necessarily the activity of the enzyme. This study identified the high-osmolarity glycerol pathway as an important process in (1) the transmission of the cold signal, (2) the regulation of expression in a subset of cold-induced genes (including cGpDH), and (3) the determination of freezing tolerance. Third, cGpDH has been identified as an enzyme that needs to be adjusted in hibernating mammals. Hibernation is not just a physiological process that lowers body temperature, it also modulates metabolism during a long period of starvation. During this starvation, new sources of glucose are required, and glycerol metabolism has been shown to provide 40–80% of this [84,85]. Glycerol is first phosphorylated by glycerol kinase to form glycerol-3-phospĥate, and is then converted by the reversible reaction of cGpDH into dihydroxyacetone phosphate (DHAP), an intermediate of gluconeogenesis and glycolysis [86]. When jerboas (*Jaculus orientalis*) enter hibernation, cGpDH can fuel catabolism by increasing activity up to two- to three-fold in various tissues, e.g., brown adipose tissue, skeletal muscle, kidneys, brain, and liver [87]. Furthermore, the liver mGpDH of hibernating ground squirrel (*Urocitellus richardsonii*) maintains better functionality at low temperature (5 °C) compared to the euthermic state form (higher affinities for glycerol-3-phosphate and NAD^+^), and displays lower perturbations when exposed to high temperature (50 °C) [88]. Another example is the black-tailed prairie dog (*Cynomys ludivicianus*); muscle cGpDH in this hibernator is structurally less rigid and maintains better functional integrity over a wide range of temperatures, has greater stability at high temperature, and is less sensitive to chemical denaturation compared to this enzyme in rabbits [86]. These specific properties may grant sustained enzyme functions over the wide range of body temperatures experienced by animals as they undergo the hibernating season.

From these studies, it appears that mitochondrial ability to oxidize G3P should be considered in future studies on either the thermal sensitivity of mitochondrial metabolism, or the adaptive or acclimatory responses to a changing environment. Another relevant reason to include G3P in the protocol of mitochondrial respiration is to ensure a proper estimation of maximal ETS capacity. An estimation of maximal ETS capacity is critical when evaluating the relative importance of different electron entrance pathways, for example, the NADH/CI or the FADH_2_/CII pathways. Respiration rates will often be normalized by the maximal oxygen consumption allowed by the ETS. Maximal ETS activity is obtained after saturating the Q pool with electrons (providing many of the mitochondrial substrates) and adding a membrane uncoupler that allows protons to leak into the matrix from outside the inner membrane, thus bypassing ATP synthase. Adequate saturation of the Q pool is, therefore, of critical importance for accurately estimating ETS capacity. It has been observed in invertebrates that the addition of G3P substrates can substantially increase the respiration rates of mitochondria that have already been provided with NADH and succinate pathway substrates [74,75,77]; consequently, we assume that feeding only these two pathways would significantly underestimate maximum ETS capacity. Accurately assessing the relative importance of different pathways feeding reducing equivalents to mitochondria at different temperatures requires proper normalization of the maximal ETS capacity, and, therefore, G3P may be needed as a substrate. For the same reasons, G3P could be useful for acclimation and adaptation (comparative) studies.

## 5. Dihydroorotate Dehydrogenase

Dihydroorotate (Dho) catabolism is an understudied pathway. To our knowledge, there is no study on the effect of temperature on Dho entry into the ETS, and there are reasons to believe that the research community would benefit from more information on this topic. This substrate is linked with many aspects of mitochondrial and cellular metabolism, and it is present in a wide variety of organisms. Here, we are considering dihydroorotate dehydrogenase (DhoDH) in animals. Bacteria and lower eukaryotes (e.g., *S. cerevisiae*) also have a form of DhoDH, but it is located in the cytoplasm, and uses either NAD^+^ or fumarate as an electron acceptor [89,90].

The Dho pathway in animals is driven by the enzyme DhoDH, a flavin mononucleotide (FMN) protein present in the outer phase of the inner mitochondrial membrane. It is the fourth step of the de novo pyrimidine synthesis pathway, it is vital for RNA and DNA synthesis, and it is the only step of this pathway within mitochondria [91]. DhoDH removes two electrons from Dho, converting it to orotate using its redox-active FMN prosthetic group; this requires a suitable electron acceptor to efficiently transfer the electron. This electron acceptor is the Q-junction of the mitochondrial ETS, which contributes to generating the electrochemical gradient [92]. It thus provides a link between the OXPHOS process and the control of biogenesis [93,94]. Pyrimidine nucleotides also serve essential functions in the activation mechanism of sugars for posttranslational glycosylation of proteins and lipids, and for phospholipid biosynthesis [95].

The Dho pathway has gained a great deal of interest recently because of its broad immunosuppressive effect in vivo, making it a promising therapeutic target for the treatment of cancer, viral infections, and auto-immune diseases [11,96,97]. Studies employing cell bypassing coenzyme Q oxidation have suggested that the only essential function of the ETS for the growth of some cancer cells is to preserve DhoDH activity [98,99], emphasizing the importance of this pathway when conditions change. Studies on the Dho pathway indicate that DhoDH inhibition can cause mitochondrial dysfunction other than what is directly linked to this pathway. First, DhoDH inhibition can induce the expression of GDF15 [91], one of the best circulating markers of mitochondrial dysfunction [100]. Second, even though the direct contribution of DhoDH to ATP generation is only marginal in various cancer cells at baseline (5–10% of routine respiration, coupled with ATP production [101]), it can vary depending on conditions. In fact, in the study of Zhang et al. [91], specific inhibition of DhoDH in cells led to a much greater reduction in mitochondrial respiration, suggesting that other processes, additional to the DhoDH pathway, were affected. Due to the fact that only routine and uncoupled respiration was used to estimate mitochondrial respiration in a standard cell culture medium (Dulbecco’s Modified Eagle Medium, DMEM), the data gave no indication as to which specific pathways were affected by the DhoDH inhibition. Third, the impact of DhoDH knockdown included a slowing of cell growth, the partial inhibition of complex III (affecting all other respiration pathways), a decrease in mitochondrial membrane potential, and an increase in mitochondrial ROS production [102]. Using immunoprecipitation and blue native-SDS/PAGE, the same study also showed that DhoDH interacts with complexes II and III [102], again suggesting potential effects beyond the provision of electrons to the Q pool via DhoDH. Fourth, DhoDH is downregulated in oral squamous carcinoma cells [103]; the loss of functional DhoDH not only impedes the de novo pyrimidine synthesis in these cells, it also disrupts mitochondrial respiration by destabilizing mitochondrial contact sites and the cristae organizing system, as well as mitochondrial homeostasis.

Even though there is no data on the effect of temperature on DhoDH, we can expect that its thermal sensitivity will affect more mitochondrial functions than only the reduction of the Q pool. Furthermore, even if it is loosely associated with the intermembrane face of the inner mitochondrial membrane, the DhoDH structure may be significantly modified due to membrane lipid alterations, impacting its activity and promoting its binding to ubiquinone [104]. It is then likely influenced by changes in temperature because temperature affects membrane structure and composition. Moreover, the association between DhoDH and the mitochondria’s inner membrane implies that its activity should be measured using a respirometry assay with the intact mitochondria, as with complex III and IV, in order to isolate the specific contribution of DhoDH to overall oxygen consumption in the OXPHOS system [101].

## 6. Choline Dehydrogenase

Glycine betaine (GB) can be synthetized by a two-step pathway: choline → betaine aldehyde → GB [105]. In animals, some fungi, and bacteria, the first step is catalyzed by mitochondrial membrane-bound ChoDH (E.C. 1.1.99.1) [106,107,108] or soluble choline oxidase [109,110,111,112], whereas it is catalyzed by choline monooxygenase in plants [113,114]. It has been shown in rat liver mitochondria that coenzyme Q might be the primary electron acceptor for ChoDH [105]. The second step—the transformation of betaine aldehyde into GB—seems to be catalyzed by NAD-dependent betaine aldehyde dehydrogenase in all organisms (reviewed by [115]), and this can be performed in the mitochondrial matrix [116]. Synthesis and/or transport and accumulation of GB is possible in a wide range of organisms.

GB is known to be involved in many cellular and biochemical processes, including the metabolism of carbohydrates, lipids, homocysteine/methionine, and ethanol as well as in macromolecule stabilization, antioxidant activity, and protein synthesis. It acts by changing the activity of various enzymes, either by directly regulating gene expression, by changing the phosphorylation status through specific kinases, or by controlling gene expression through changes in the degree of methylation of target gene promoters (reviewed by [115]). Considering its involvement in many metabolic pathways, GB is believed to be involved in many diseases, including diabetes, Alzheimer’s, Parkinson’s, Huntington’s disease, cardiovascular diseases, nonalcoholic steatohepatitis, and kidney diseases (reviewed by [115]). Interestingly, even though mutations in genes associated with choline transporters and choline metabolism enzymes can cause the childhood onset of severe neurologic and metabolic diseases, no diseases have been associated with ChoDH mutations in humans [117]. In fact, mice that have undergone ChoDH knockout are viable and overtly healthy, although both males and females are infertile (https://www.mousephenotype.org/ from [11], accessed on 1 April 2022). In contrast, choline is an essential nutrient in some conditions, and its deficiency is associated with liver diseases (reviewed by [11]).

Many bacteria, plants, and animals will accumulate glycine betaine (GB) under abiotic stress conditions [118,119]. In plants, betaine is synthesized and accumulated as an osmoprotectant against salt and temperature stresses (reviewed by [120]). Transgenic plants engineered with bacterial enzymes that synthesize glycine betaine showed enhanced tolerance towards various environmental stresses, including freezing, high temperatures, and hypersalinity [106]. Furthermore, tobacco cells showed an increase in thermotolerance following the overproduction of proline while exposed to high temperatures [121]. *Synechococcus* sp. and *Arabidopsis thaliana* transformed with genes from the soil bacteria *Arthrobacter globiformis* that encode choline oxidase (codA) have demonstrated improved tolerance to both high and freezing temperatures [122,123,124]. Enhanced tolerance to low temperatures has been also observed during germination of transgenic *A. thaliana* seeds transformed with codA [125,126]. The cryoprotective effect of GB might come from its ability to stabilize tertiary protein structures and prevent, or reverse, the disruption of tertiary protein structures caused by non-compatible (perturbing) solutes [127]. Increased GB levels in plants also cause a significant increase in freezing tolerance [128,129,130,131]. Plants facing thermal stress accumulate osmoprotective solutes with a high N content, such as quaternary ammonium compounds (QACs) (reviewed by [132]). The most common QACs are glycine, betaine, and choline, and they notably accumulate under both low [133] and high [134,135] temperatures. Choline is a fundamental precursor of phosphatidylcholine, which is a dominant constituent of eukaryote membrane phospholipids, and a large proportion of free choline is released into the cytoplasm during environmental stress [136,137], potentially contributing to the protective role of choline during thermal stress.

Choline dehydrogenase therefore appears to be mainly involved in providing metabolites that protect plants from stressful conditions linked to temperature changes. However, it is still unknown whether similar mechanisms occur in animals. It is known that ChoDH is associated with the modulation of mitophagy in mammalian cells [138]. This study suggested that ChoDH is required for the recruitment of SQSTM1 and LC3, which are essential for PARK2-mediated mitophagy. Knowing that mitophagy promotes the maintenance of homeostasis and is sensitive to oxidative stress and [139], and that temperature significantly affects oxidative stress in invertebrates [140,141] and fish [4], it would be worthwhile to explore the evolution of ChoDH in the context of thermal adaptation and acclimation.

## 7. Proline Dehydrogenase

Proline can be a fuel source in bacteria, protists, plants, and animals (reviewed by [142]). In eukaryotic cells, proline dehydrogenase (ProDH) is located in the inner mitochondrial membrane, and catalyzes the oxidation of L-proline into pyrroline-5-carboxylate. The enzyme ProDH shuttles electrons directly into the ETS Q-junction, or to oxygen [142]. In mammals, ProDH has been shown to support cell respiration under acute nutrient stress [143,144]. Pyrroline-5-carboxylate produced by ProDH can be hydrolyzed nonenzymatically to glutamic semialdehyde, which is further oxidized to glutamate during an NAD^+^-dependent reaction catalyzed by pyrroline-5-carboxylate dehydrogenase (P5CDH). Another route for pyrroline-5-carboxylate is conversion into ornithine, which is connected to the urea cycle [145]. Glutamate produced by P5CDH can be deaminated by alanine aminotransferase, or by glutamate dehydrogenase. This leads to α-ketoglutarate, which acts as a sparker metabolite that replenishes the citric acid cycle, feeding reducing equivalents from complexes I and II to the ETS, and maintains a higher potential for pyruvate oxidation (reviewed by [11,68]). The metabolism of proline is also closely linked with the pentose phosphate pathway in animals [145,146].

The importance of proline catabolism in cellular homeostasis is underscored by its link with many other metabolic pathways (ETS, CAC, pentose phosphate pathway, urea cycle). For example, an association between ProDH and genotoxic, inflammatory, and metabolic stress has been suggested [143]. When a cell faces variations in its internal and environmental context, programmed cell death can be induced through ProDH [147,148,149]. The induction of ProDH expression by p53 leads to increased proline oxidation, reactive oxygen species formation, and the induction of apoptosis; however, proline dehydrogenase may also be required to ensure proline protection against peroxide-induced cell death [150]. It has been suggested that proline and ProDH help to maintain NADPH, which is required to prevent and/or attenuate oxidative stress. The same authors [150] observed that ProDH activates Akt during H_2_O_2_ exposure, which inhibits FoxO3a and blocks cell death. Proline metabolism has also been associated with lifespan in nematodes [151] and yeast [152].

Proline can be a direct electron donor to the Q-junction (independent of complex I and II activity) or an indirect electron donor in the citric acid cycle. It was shown that most proline-induced mitochondrial respiration is independent of complex I in insects [153], mouse liver mitochondria, a human cell model, and cancer cells [143]. However, succinate inhibits ProDH in a noncompetitive way and prevents ROS generation by ProDH, suggesting that there is cross-talk between proline and succinate in terms of their roles in partially controlling mitochondrial respiration and their different signaling pathways associated with stress responses [143].

Sporadic data on the proline oxidation pathway seem to show that the activities and functions of ProDH are highly diverse and depend on the environmental, cellular, and phylogenetic contexts, even within a group of closely related species (reviewed by [68]). Proline can be used as a main fuel, as a co-substrate, or simply as a sparker metabolite [68,153]. An exceptional reliance on proline as an energy substrate has been observed in various species of invertebrates, such as some flying and blood-feeding insects [153,154,155,156,157,158,159]. It was shown that proline oxidation coupled with respiration is more prominent in female mosquitoes (*Aedes* *aegypti*) compared to males, and that blood-feeding is also restricted to females [73]. In at least one bivalve species (*Mercenaria mercenaria*; [160]), proline is the preferred substrate of the mitochondria. In some other species of insects (e.g., beetles) and in cephalopods, proline is used not as a main substrate, but as a sparker substrate, working in combination with carbohydrates and providing additional intermediates to the TCA cycle to support high activity rates (reviewed by [161]).

Considering the limited information on the utilization of proline as a mitochondrial substrate by eukaryotes, the impact of temperature on proline metabolism, as well as its importance in temperature adaptation or acclimation, are still open fields to explore. Some studies have shown that proline accumulates prior to overwintering in gall fly larva (*Eurosta solidaginis*) [162] and in southwestern (*Diatraea grandiosella*) and European (*Ostrinia nubilalis*) corn borers [163]. Proline accumulation depends on alterations of ProDH or proline oxidase functions. Changes in proline metabolism have been related to survival when facing thermal stress in the parasite *Trypanosoma cruzi*, the etiological agent of Chagas disease [164]. The involvement of proline in resistance to high temperature stress has been linked to the extreme thermal resistance—up to 70 °C—of the enzyme Δ^1^-pyrroline-5-carboxylate reductase, which catalyzes proline biosynthesis [165]. A conclusive metabolomics analysis of CI-deficient muscle has shown that proline oxidation, along with other substrates, such as succinate, glycerophophate, and fatty acids, can compensate for complex I deficiency by feeding electrons directly into the ETS Q-junction [166]. Modulation of rel ProDH content and activity could, therefore, be an adaptive response to compensate for the alterations in key electron transport processes (for example CI) following changes in environmental conditions, including temperature.

Proline metabolism has also been linked with tolerance to temperature changes in plants and fungi. Many plants under thermal (cold and heat) stress accumulate osmoprotective solutes with a high N content, such as choline and proline (e.g., [132,133,134,135,167,168]). Proline content has even been identified as one of the major desirable criteria for screening heat tolerance in wheat [169]. Proline acts to protect cell structure [170] and encourages growth following the stress period [170,171,172]. In creeping bentgrass (*Agrostis stolonifera* L.), a close association between cold stress tolerance and the proline-associated pentose phosphate pathway has been established [173]. Enhanced proline concentration has also been observed when facing high temperature stress in the fungus *Aspergillus flavus* [174]. More research is needed to understand the role of this pathway in tolerance to temperature variation in animals.

## 8. Sulfide:Quinone Oxidoreductase

Hydrogen sulfide (H_2_S) is an environmental toxin; at high concentrations, it can inhibit mitochondrial respiration through tight binding to cytochrome *c* oxidase [175,176,177] and mitochondrial depolarization [178]. To prevent this poisoning, sulfide:quinone oxidoreductase (SQOR) couples H_2_S oxidation to ubiquinone reduction, providing electrons to complex III (see [179] for a review). When present at lower concentrations, H_2_S can play a signaling role in the cardiovascular, central nervous, and gastrointestinal systems of mammals [180]. Mitochondria have been identified as a major target. For example, sulfidration of ATP synthase at low concentrations increases this enzyme’s activity in human-derived cell lines [181]. These concentration-dependent antagonistic impacts of sulfide on mitochondrial functions might require the tight regulation of its concentrations. The adjustment and control of SQOR activity could, therefore, respond not only to insure detoxification, but also to modulate sulfide concentration in the range of its signaling functions. It remains to be explored to what extent SQOR is involved in the adaptation to sulfide-rich and fluctuating environments, such as the deep sea and ocean sediments, or if this enzyme has any adaptive functions during temperature fluctuations. A recent study of the transcriptomic response of deep-sea mussels (*Gigantas platifrons*) to exposure to high sulfide concentrations revealed upregulation of oxidative phosphorylation and sulfide oxidation, suggesting plasticity in sulfide detoxification capacity [182]. In other studies on zebrafish (*Danio rerio*), it has been observed that SQOR expression is modulated by temperature [183,184,185,186]. These observations indicate a role of SQOR in adaptive responses to thermal stress, and call for further investigations.

## 9. Conclusions

To understand the effect of temperature on metabolism, it is first important to acquire a good picture of the preferred mitochondrial substrates and pathways specific to the species, tissues, and thermal conditions. Considering the number of substrates and pathways involved, determining the impact of temperature on mitochondrial metabolism thus becomes very complex. Determining how persistently preferred pathways are used requires respiration measurements for each substrate under various temperature conditions. The maximum catalytic capacity of mitochondria can also be informative. For this, we need to verify that the Q-junction is saturated at the mitochondria’s highest capacity by ensuring an adequate provision of electrons to the ETS. The sensitivity of mitochondrial respiratory control can also be explored. The impact of adding an uncoupler could reveal to what extent OXPHOS is controlled by the phosphorylation system (i.e., ATP synthase, adenine nucleotide translocase, phosphate carrier), which varies greatly among species [10,187,188]. A more detailed approach to examine respiratory control would be to estimate the control strength of different steps using inhibitors of specific pathways (see [74]). Most studies on sensitivity to environmental conditions have focused on respiration rates usually measured by high-resolution respirometry, but respiration rate and ATP synthesis are not the only outputs of mitochondria; the management of ROS production and buffering also become major concerns [189,190]. Finally, we should be aware of the consequences that changes in environmental conditions have on the efficiency of mitochondrial respiration, i.e., the amount of ATP synthetized per unit of oxygen consumed [191,192]. All these considerations for studying mitochondrial sensitivity are also valuable for acclimation and adaptation studies.

To further forecast the ability of ectotherms to adapt to a changing environment, we should be aware of highly sensitive traits of physiological and biochemical phenotypes that might impose limitations following change. In view of the central role of mitochondria in bioenergetics and the regulation of different pathways, it is likely that mitochondrial adaptation is a key feature determining the ability to colonize new thermal habitats. The fundamental question is which parts and pathways associated with the mitochondria can impose constraints in a changing environment. Knowledge of the weak areas will allow the exploration of their variability, heritability, and, therefore, evolvability. The first step required to identify these weak traits is to better portray the thermal sensitivity of the key pathways and steps identified in this review. This represents a major enterprise that calls for the involvement of many more comparative physiologists and biochemists than the few already active this field.

a = 1(1)
